# Artificial intelligence in radiology: Radiologist’s adversary or comrade?

**DOI:** 10.12669/pjms.41.1.11391

**Published:** 2025-01

**Authors:** Ali Mansoor

**Affiliations:** Dr. Ali Mansoor, MBBS, FCPS, FRCR Department of Radiology, Ameer ud Din Medical College/Post Graduate Medical Institute/Lahore General Hospital, Lahore, Pakistan. Email: dr.alimansoor@hotmail.com

It may feel a bit surprising amid the boom of artificial intelligence (AI) that we are currently experiencing that the term was first used as long ago as 1956 at a conference held in Dartmouth.^1^ Today, we find ourselves surrounded by a multitude of AI applications in our daily lives - the moment we wake up to use the facial recognition feature of our smartphone to unlock it to use Google Maps for navigation, use Google Assistant and Siri to assist us on our smart devices to chatbots that assist on websites – the list seems to be unending. Beyond this, AI lends itself to numerous professional applications including the field of medicine.

Radiology has always been at the forefront of technological advances in the field of medicine and AI is no exception. Radiology data in the form of radiology reports and images archived in Picture Archiving and Communication System (PACS) provide the perfect substrate for machine learning and are therefore ideal for developing AI tools. This has led to a boom of research on the use of AI in radiology. On one hand, this promises to offer benefits to patients and healthcare systems, but on the other hand, it has created panic among radiologists who may see it as their ‘replacement’.

AI has many interpretative uses in imaging – neuro radiology, breast, thoracic imaging, and musculoskeletal system are some of the specialities where AI applications are already in use.^2^ A recent speciality-specific meta-analysis suggested that deep learning algorithms generally have a high and clinically acceptable diagnostic accuracy in identifying diseases on imaging.^3^ Indeed, there are situations where this may even surpass the radiologist’s abilities. In screening examinations where there are low chances of detecting an abnormality, observer fatigue may limit radiologist’s interpretative skills but AI is beyond such errors.^4^ As the radiology workload continues to increase amid a shortage of workforce, an average radiologist must interpret one image every 3 to 4 seconds, making him prone to make errors under such restrained conditions.^5^ AI has no such limitations. It was due to factors like this that claims were made of AI replacing radiologists in the future, ending radiology as a thriving speciality.^6^ Adding fuel to the fire, AI applications in radiology extend beyond mere interpretation of images. Image production and quality control, improving radiology workflow, business applications, research applications and radiology education are some of these.^7^

All this would make one believe that AI is the perfect replacement for radiologists. The reality is, however, different. AI faces intrinsic challenges for application in the field of radiology. Biological systems are very complicated and imaging data is very heterogeneous both at individual and population level thereby implying that it may be very difficult to define the threshold to distinguish normal from abnormal in nominal data. The situation may become more difficult for rarer diseases where data may be insufficient to train AI leading to a risk of overfitting.^4^ This may be compounded further by proprietary interests – it is not necessary that every institution would be ready to share their data for AI development. Validity of application may be another problem where the same results and thresholds would not be necessarily relevant to a particular population or ethnic group. Apart from these intrinsic limitations, there are external obstacles as well. Stringent regulations imply that the use of AI as a standalone technique requires extensive research which may be too expensive to be cost effective. Patient acceptability is another issue – being managed by an AI tool alone without any human oversight is unlikely to be a popular option. Legal implications only add to this challenge – the developers must face financial litigations when AI makes any mistake.

The solution to all these problems lies in developing a harmonious relationship between AI and radiologists – a situation whereby advantages can be taken of each one’s strengths and use them to make up for other’s limitations. It is highly improbable that any radiologist enjoys the tasks of measuring lesions, doing segmentation or going through records to gather pertinent clinical information about the patient. These tasks can be left to AI. Similarly, AI tools can be used as screening tools for studies in long queues to categorize which studies are to be read first, increasing sensitivity at the cost of accuracy in life-threatening conditions and at the same time alerting the radiologists to prioritize their interpretation.^4^ Same prioritizations can also be done in screening scans – separating positive cases from negative ones, aiding the radiologist to interpret those first before observer fatigue sets in. This, and other such tasks, performed amicably by AI would lead to more time being available to radiologists to do what they are better at in comparison to AI – participation in multidisciplinary team meetings and more patient interactions to explain the results of imaging as well as taking primary responsibility of patient care in case of interventional procedures.^8^

In conclusion, it is the need of the hour for radiologists to let go of their fears and embrace AI and use it to their strength instead of closing their eyes to this reality or trying to oppose this change. The same goes for the advocates of AI – they must try to develop and implement the AI tools as an adjunct to human resource rather than a replacement because, within the current state of affairs, an AI tool as the sole decision maker for human life will not be presently acceptable to any of the stakeholders. If a friendly relationship can be built between radiologists and AI, this camaraderie would be beneficial for the patients as well and lead to ultimate improvement in healthcare.
